# From package to practice: standardizing surgical equipment to increase efficiency and access

**DOI:** 10.3389/fpubh.2026.1957552

**Published:** 2026-10-14

**Authors:** Nardeen B. Dawood, Priyansh Nathani, Gabriella Y. Hyman, Adriana Velazquez Berumen, Anita Gadgil, Nobhojit Roy, Nakul P. Raykar

**Affiliations:** 1 WHO Collaboration Center for Emergency, Critical and Operative Care, Program in Global Surgery and Social Change, Harvard Medical School, Boston, MA, United States; 2 Department of General Surgery, New York University Langone, New York, NY, United States; 3 WHO Collaboration Center for Emergency, Critical and Operative Care, Program for Global Surgery and Trauma, The George Institute for Global Health, Delhi, India; 4 Department of Plastic and Oral Surgery, Boston Children's Hospital, Boston, MA, United States; 5 Former WHO Team Lead Medical Devices and In Vitro Diagnostics, Geneva, Switzerland; 6 Department of Global Public Health, Karolinska Institutet, Stockholm, Sweden; 7 Department of Surgery, Brigham and Women's Hospital, Boston, MA, United States

**Keywords:** global surgery, operating room efficiency, surgical instrument sets, surgical standardization, Universal Health Coverage

## Abstract

Surgical conditions account for approximately one third of the global burden of disease. In 2025 at least 160 million additional operations per year were needed to meet this need, which is greatest in low- and middle-income countries (LMICs). Standardizing surgical packages is a key step toward improving operating room (OR) efficiency and expanding surgical care delivery across high-, middle-, and low-resource settings. Yet in low-resource settings, surgical equipment procurement often remains fragmented, non-standardized, and driven by facility-level *ad-hoc* processes rather than procedure-based planning. A universal standard could support high-quality surgical care that is both nationally scalable and internationally consistent. The World Health Organization's (WHO) service package delivery and implementation (SPDI) tool is a viable candidate for such standardization of surgical instrument sets as it already covers nearly all essential surgeries identified by existing normative frameworks, making it a strong foundation for universal surgical package standardization. National implementation of reduced, standardized surgical sets could extend demonstrated single-center efficiency gains by improving OR efficiency and increasing case volume, supporting case reproducibility and more consistent care, and making supply chains more reliable, cost-effective, and environmentally sustainable. Pre-defined packages lend themselves to rapid, cost-effective scale-up of supply chains, while minimized sets can also lower the carbon emissions associated with surgical care. Ministries of health, hospital networks, and practitioners at the facility level may consider the WHO's UHC Compendium as a foundation for expanding access to safe, high-quality surgical care.

## 1 Introduction

Surgical conditions are thought to account for 28%−32% of the overall global burden of disease (1). In 2025, at least 160 million additional operations per year were needed to meet this burden (2). Health systems can avert associated mortality by providing patients with safe, timely, and affordable surgical care. To deliver this standard of care, they must maintain the necessary infrastructural and operational capacity. Surgical service delivery relies on multiple interrelated factors, including expedient patient transport, appropriate diagnostics, operative room availability, surgical equipment and supplies, human resources, intensive care capabilities, and other essential services. In addition to workforce and infrastructure, health systems must also operationalize processes that, when optimized, ensure maximal service delivery and improve patient outcomes.

Standardizing surgical packages is a key step to improving operating room (OR) efficiency and expanding surgical care delivery across high-, middle-, and low-resource settings. Surgical packages are itemized descriptions of the supplies required for a given operation, including the diagnostics, surgical medical equipment, consumables, and sterile surgical instruments. The creation of optimized surgical sets, also known as surgical trays, has been protocolized at many independent centers, yet universally standard sets that transcend geographic, rural-urban, and resource-diverse contexts have remained largely aspirational. A universal standard could support high-quality care that is nationally scalable and internationally consistent. In many low-resource settings, procurement is fragmented, non-standardized, and driven by facility-level *ad-hoc* processes rather than procedure-based planning. As a result, what is available in the operating room may reflect what was requested or procured sporadically, rather than what is required for safe and efficient care.

Globally transparent and rational procurement practices can be enhanced by standardized WHO tools. The Universal Health Coverage Compendium (UHC-C) houses an extensive catalog of medical and surgical packages. Its service package delivery and implementation (SPDI) tool allows user countries to select packages for inclusion in their national health service plans.^1^ The medical devices (i.e., diagnostics, medical equipment and surgical instruments) are taken from the WHO medical devices information system (MeDevIS) and the surgical sets listed in the WHO medical devices packages (MeDevPacks). One example amongst its 91 surgical instrument sets is the non-consumable laparotomy instrument set, which outlines the specific technical characteristics (e.g., shape, angle, edge, material) and quantities of forceps, needle holders, retractors, scissors, and other instruments. The MoSCoW prioritization framework was used to streamline the essential instruments that are required for a given procedure, whereas the “Must Do, Should Do, and Can Do” framework described below was used to identify which procedures are the highest priority for first-level care.

## 2 Approach and outcomes

At a minimum, a global standardization tool must adequately cover essential surgeries. Low-resource health systems often require clinicians to prioritize a select group of essential surgical procedures in the first-level hospital setting. Two frameworks that identify priority operations are: (1) The third edition of the Disease Control Priorities' (DCP3) essential surgeries and, (2) The Lancet Commission on Global Surgery's (LCoGS) “Must Do, Should Do, and Can Do” operations (3, 4). To test the coverage of the SPDI tool, each procedure from both frameworks was queried in the SPDI platform to assess whether an SPDI entry and surgical instrument set were present. Twenty-nine of 44 DCP3 procedures had entries with embedded instrument sets. Of the LCoGS-identified procedures, 4/5 “must do,” 6/8 “should do,” and 3/8 “can do” procedures had entries with embedded instrument sets (Table 1).

**Table 1:** The WHO UHC-Compendium's surgical instrument sets and their alignment with normative essential surgical procedure lists.

WHO UHC-Compendium: Surgical Instrument Sets‡		
Abdominal surgery set^*^†	Functional endoscopic sinus surgery (FESS) set	Orthopedic surgery set†
Amputation set†	Gastrointestinal surgery set	Pediatric surgery set†
Biopsy–gynecology set	General catheter placement instrument set†	Pelvic exenteration set
Bone marrow aspiration set	General surgery set^*^†	Plastic and reconstructive surgery set^*^
Bone marrow transplantation set	Glaucoma surgery set	Preperitoneal pelvic packing set
Bone wiring and kirschner set	Gynecology and kidney surgery set^*^†	Prostatectomy set
Bronchoscopy set	Hydrocelectomy set†	Prosthetic joint replacement set
Burr hole set^*^†	Hysterectomy, abdominal set†	Retinoblastoma cryotherapy set
Cataract surgery set^*^†	Hysterectomy, vaginal set†	Retinoblastoma laser therapy set
Cervical canonization set	Incision and drainage surgery set†	Septoplasty set
Cervical electrosurgical procedure (LEEP/LLETZ) set	Internal fracture fixation set^*^†	Skin grafting set†
Cervical surgery set	Keratoplasty surgery set	Spinal fusion surgery set
Colposcopy set	Lacrimal surgery set	Stapedectomy set
Corneal surgery set	Laminectomy set	Strabismus surgery set
Deep brain stimulation set	Laparotomy, cesarean surgery set^*^†	Surgical spinal decompression set
Delayed primary closure (DPC) set	Laryngoscopy set	Surgical spinal fixation set
Dental diagnostic set	Liver or kidney transplant set	Suture set†
Dental extraction set†	Lung surgery set	Thoracic surgery set^*^
Dental surgery set†	Manual vacuum aspiration set†	Tonsillectomy and adenoidectomy set
Dilation and curettage set^*^†	Mastectomy set^*^	Tracheostomy set
Ear, nose, and throat (ENT) surgery set^*^†	Mastoidectomy set	Trachoma surgery set†
Endocervical curettage set	Microsurgery–advanced set	Traction set
Endoscopy surgery set^*^	Microsurgery–basic set	Tympanoplasty set
ENT surgical exploration set	Microsurgery set	Urethral sounding set
Enucleation and evisceration set	Minor surgery set^*^†	Urology surgery set^*^†
Escharotomy set†	MRI-guided cerebral biopsy set	Vaginal surgery set^*^
External fracture fixation set^*^†	Neurosurgery set^*^	Vascular surgery set
Extracapsular cataract extraction (ECCE) set^*^†	Open lobectomy set	Ventriculoperitoneal shunt set†
Eyelid surgery set†	Ophthalmic trauma set	Vitrectomy set
Foreign body removal set		Vitreoretinal surgery set
		Wound debridement–surgical set^*^†

^*^Surgical instrument set accommodates a “Must Do, Should Do, or Can Do” procedure as outlined by the Lancet Commission on Global Surgery.

†Surgical instrument set accommodates an “essential surgery” as outlined by the Disease Control Priorities, Third Edition.

‡Listed in the UHC Compendium SPDI tool, WHO Medical Devices Information System (MeDevIS), and in the sets of the Medical Devices Packages (MeDevPacks) system.

For procedures lacking a SPDI entry with an associated instrument set, the authors conducted a qualitative review and assigned the procedures to one of threecategories:

1. No SPDI entry exists, but coverage is achieved by an alternative existing instrument set [e.g., superficial abscess drainage (incision and drainage surgery set), drainage of dental abscess (dental surgery tray)]; *n* = 10.
2. An SPDI entry exists with an embedded surgical set that is not yet accessible due to a pending website update [e.g., dilation and evacuation (vacuum aspiration set)], *n* = 2.
3. An SPDI entry exists but lacks an embedded surgical set; coverage is achieved by an alternative existing surgical set [e.g., tubal ligation (gynecology set), colostomy creation (abdominal surgery set)], *n* = 10.

Apart from a single procedure, uretero-renoscopy, all essential surgeries identified by Disease Control Priorities 3 and the Lancet Commission on Global Surgery were either explicitly built into the SPDI tool or indirectly included in existing alternative surgical sets per qualitative evaluation. This breadth of coverage positions the SPDI tool as a strong candidate for universal standardization.

## 3 Discussion

Universal standardized packages are expected to offer the benefits of improving operative efficiency, reproducibility, cost-effectiveness, and environmental sustainability. Ultimately, these advantages are expected to support high-quality patient care and more reliable supply chains. Importantly, standardized packages should function as minimum system-level standards rather than fixed or exhaustive requirements, with surgical care providers retaining discretion to request clinically justified additions.

### 3.1 Improve operative room efficiency to increase case volume

Standardized surgical packages are expected to make operating rooms more efficient. Because the UHC-C surgical sets are optimized in both composition and quantity, instruments can be strategically allocated across sets without compromising safety. This more judicious distribution of instruments enables the following proven approaches to increase efficiency: streamlining tray assembly and sterilization, expediting operating room turnover, and reducing intra-operative time (5). Evaluation of optimized trays for four head and neck procedures demonstrated a 58% to 66% (*P* < 0.05) decrease in assembly time and 26% to 37% (*P* < 0.05) decrease in OR setup time (6). Using a Lean 5S methodology (sort, simplify, sweep, standardize, self-discipline), setup time for minimally-invasive spine surgery decreased by 37% (13.1–8.2 min, *p* = 0.0015) (7).

Efficient operative rooms with high surgical throughput are likely to reduce surgical waiting times and delays in essential surgical care. National implementation of reduced surgical sets could extend these single-center efficiency gains. Thus, these efficiency gains could support improved surgical access beyond individual patients to entire populations.

### 3.2 Facilitate case reproducibility to ensure quality

Operative consistency may promote outcome consistency, an indicator of high-quality care. When centers standardize surgical equipment, surgical preparation can become more reproducible. Adopting the UHC Compendium's packages into operative practice is expected to make the medications, team compositions, consumable supplies, instruments, and OR equipment more consistent across cases. This may have additional use cases in conflict zone areas where inventories are often disrupted. Making operations reproducible may also simplify training particularly in remote settings where basic surgery is frequently task-shifted to generalist clinicians or in academic settings where residents are acquiring new operative skills.

Furthermore, standardized surgical processes, such as the WHO surgical safety checklist, have been associated with lower postoperative complications and peri-operative mortality (8). In the same vein, standardized surgical packages may reduce workflow variability and opportunities for error, although their direct effects on patient outcomes require further evaluation.

### 3.3 Make supply chains reliable, cost-effective, and environmentally sustainable

Standardizing surgical equipment may facilitate rapid, cost-effective, and environmentally sustainable scale up of surgical service delivery. Optimized surgical sets can help health ministries to mitigate widespread supply chain shortages. A necessary first step is to define a minimum service standard for essential equipment. The UHC Compendium established minimum standards using expert clinician review. The program harnessed the technical discretion of 20 surgical faculty members across variable resource contexts to determine the optimal lean composition of each surgical package. Standard, lean packages can then streamline supply chain processes. Thus, equipment availability may not actually be a luxury, but the product of a systematized standardization strategy.

Minimizing surgical sets can rationalize instrument use and reduce health system costs (9). Such cost savings may reduce financial pressure on health systems. Whether they translate into lower costs for patients requires further evaluation.

Reducing surgical equipment lowers the environmental burden from consumable use and sterilizing non-consumable instruments. Like many other single-center studies, a tray reduction program in the UK found that reducing surgical trays by about 50% for pediatric open herniotomy was associated with an annual reduction of approximately 384 kgCO2 emissions (10). If adopted at scale, standardized surgical sets could contribute to reductions in health care system carbon emissions.

### 3.4 Change as a downhill battle

Change is always met with resistance. Challenging surgical care providers to deviate from the trays they are accustomed to is no exception. However, it is worth clarifying what standardization does and does not mean in this context. Standardized packages are primarily a system-level intervention designed for health ministries, hospital administrators, and supply chain planners to define minimum standards, streamline procurement, and expand access.

They are not meant to supersede the clinical judgement of individual surgical care providers, who must retain flexibility to adapt standardized packages to particular patients, procedures, or clinical situations.

However, at the system level, surgical care providers may very well covet having access to consistently complete trays, expedited OR turnover times, and a reduced carbon footprint more than they grieve personalized surgical trays.

## 4 Conclusion

Standardized surgical packages are expected to promote OR efficiency, case reproducibility, and high-quality patient care. Pre-defined sets of medications, instruments, and operative equipment lend themselves to rapid, cost-effective scale-up of supply chains. Minimized sets can also lower the carbon emissions associated with surgical care. Ministries of health, hospital networks, and practitioners at the facility level may consider the WHO's UHC Compendium as a foundation for surgical care expansion, while allowing adaptation to local context and individual clinical judgement.

## Data Availability

The datasets presented in this study can be found in online repositories. The names of the repository/repositories and accession number(s) can be found at: World Health Organization: Universal Health Coverage Compendium. https://uhcc.who.int/uhcpackages/package/groups?packageId=735&amp;viewMode=flat.

## Footnotes

1 https://uhcc.who.int/uhcpackages/package/groups?packageId=735&viewMode=flat.
